# Age-Dependent Risk of Long-Term All-Cause Mortality in Patients Post-Myocardial Infarction and Acute Kidney Injury

**DOI:** 10.3390/jcdd12040133

**Published:** 2025-04-03

**Authors:** Keren Skalsky, Mashav Romi, Arthur Shiyovich, Alon Shechter, Tzlil Grinberg, Harel Gilutz, Ygal Plakht

**Affiliations:** 1 Department of Cardiology, Rabin Medical Center, Petach Tikva 4941492, Israel; kskalsky@gmail.com (K.S.); ashiyovich@bwh.harvard.edu (A.S.); alonshechter@gmail.com (A.S.); tzlilgrin@gmail.com (T.G.); 2 Faculty of Medicine, Tel Aviv University, Tel Aviv 6997801, Israel; 3 Faculty of Health Sciences, Ben-Gurion University of the Negev, Beer Sheva 8410501, Israel; mashav1234@gmail.com (M.R.); gilutz@bgu.ac.il (H.G.); 4 Neonatal Intensive Care Unit, Sheba Medical Center at Tel HaShomer, Ramat Gan 5262000, Israel; 5 Division of Cardiovascular Medicine, Department of Medicine, Brigham and Women’s Hospital, Harvard Medical School, Boston, MA 02115, USA; 6 Department of Cardiology, Smidt Heart Institute, Cedars-Sinai Medical Center, Los Angeles, CA 90048, USA; 7 Department of Emergency Medicine, Soroka University Medical Center, Beer Sheva 8410101, Israel

**Keywords:** acute kidney injury, kidney disease, myocardial infarction, survival

## Abstract

Objectives: We aimed to investigate the association between acute kidney injury (AKI) and the risk for long-term (up to 10 years) all-cause mortality among elderly compared with younger patients following an acute myocardial infarction (AMI). Methods: This study was a retrospective analysis of the Soroka Acute Myocardial Infarction registry and covered the years 2002 to 2017. It included patients diagnosed with an AMI who had a baseline estimated glomerular filtration rate (eGFR) greater than 60 mL/min/1.73 m^2^ and serum creatinine measurements available during hospitalization. The patients were stratified by age: elderly (aged 65 years or older at admission) and younger. In each stratum, two groups were defined based on the presence of an AKI. The survival approach (Kaplan–Meier survival curves, log-rank test and Cox regressions) was utilized to estimate and compare the probability of long-term (up to 10 years) all-cause mortality in each group. Results: Among the 10,511 eligible patients, which consisted of 6132 younger patients (58.3%) and 4379 elderly (41.7%), an AKI occurred in 15.2% of cases, where the elderly patients experienced a higher incidence than the younger patients (20.9% vs. 11.2%, *p* < 0.001). The presence of an AKI significantly increased the risk of death in both age groups, with the association being stronger among the younger patients (AdjHR = 1.634, 95% CI: 1.363–1.959, *p* < 0.001) than among the elderly (AdjHR = 1.278, 95% CI: 1.154–1.415, *p* < 0.001, *p*-for-interaction = 0.020). Conclusions: An AKI following an AMI was associated with a high risk for long-term all-cause mortality in both age groups, with a stronger association among younger patients.

## 1. Introduction

An acute kidney injury (AKI) is a common complication following an acute myocardial infarction (AMI) and is associated with increased morbidity and mortality [1]. The reported incidence of an AKI in this context varies between 26% and 36.6%, depending on the study population and series analyzed [2,3]. The pathophysiological mechanisms leading to an AKI after an AMI typically involve a combination of hemodynamic fluctuations, inflammation and oxidative stress. These, in turn, cause both direct parenchymal injury and low renal perfusion [1,4,5,6]. Previous research demonstrated that an AKI occurring post-AMI is independently linked with increased morbidity and mortality. Patients who develop this condition are at a higher risk for both short- and long-term complications, including chronic kidney disease (CKD), end-stage kidney disease (ESKD) and major adverse cardiovascular events (MACEs) [7,8,9,10,11,12]. Age is a key factor influencing the risk of an AKI, with older patients being more susceptible to the condition [13,14]. Age alone is an independent predictor of MACEs, and those above 65 years of age face a higher risk of long-term mortality compared with younger patients [15,16,17]. This study focused on assessing the long-term mortality in elderly patients who develop an AKI following an AMI in comparison with younger patients.

## 2. Materials and Methods

### 2.1. Study Population

This study used data from the Soroka Acute Myocardial Infarction (SAMI) registry, which includes all consecutive hospitalizations for AMIs at the Soroka University Medical Center (SUMC), Israel, between 1 January 2002 and 31 October 2017 [7]. The analysis included adult Israeli citizens (aged ≥ 18) who were hospitalized for an AMI at the SUMC and were discharged alive. Additional inclusion criteria were an estimated glomerular filtration rate (eGFR) greater than 60 mL/min/1.73 m^2^ at the time of admission and the availability of at least two serum creatinine (Cr) measurements within the first seven days of hospitalization. The first serum creatinine measurement recorded within 24 h of admission was considered the baseline value for the analysis. The patients were stratified by age: elderly (aged 65 years or older at admission) and younger. In each stratum, two study groups were defined based on the presence of an AKI. An AKI was defined according to standard criteria, namely, an absolute increase in creatinine of ≥0.3 mg/dL (≥26.5 μmol/L) within 48 h or a relative increase in creatinine to ≥1.5 times the baseline level within seven days [9,18].

This study was conducted in compliance with the Declaration of Helsinki and received approval from the Soroka Institutional Review Board. Due to the retrospective nature of this study, the requirement for informed consent was waived.

### 2.2. Follow-Up and Outcome

This study’s follow-up period extended from the hospital discharge until death or 31 July 2023, whichever occurred first. The primary outcome of interest was the all-cause mortality up to 10 years following the hospital discharge. The data on deaths were retrieved from the Israeli Ministry of the Interior’s Population Registry.

### 2.3. Data Collection and Definitions

The diagnosis of an AMI was based on ischemic symptoms and/or signs combined with a characteristic rise and fall in cardiac biomarker levels, as per the applicable Universal Definition of Myocardial Infarction [19]. The International Classification of Diseases, Ninth Revision, Clinical Modification (ICD-9-CM) code 410 was utilized to identify patients hospitalized for an AMI.

Baseline comorbidities were recorded using ICD-9-CM codes, as documented by the attending medical team during the hospital stay (Supplementary Table S1). Beyond the ICD-9-CM criteria, obesity was characterized by a body mass index (BMI) of 30 kg/m^2^ or higher [20] and dyslipidemia was defined as low-density lipoprotein (LDL) levels equal to or exceeding 100 mg/dL within the 12 months preceding or following hospitalization [21]. Anemia was identified by a blood hemoglobin concentration below 13 mg/dL in men or under 12 mg/dL in women [22].

Obstructive coronary artery disease was determined by identifying a vessel stenosis of ≥70% on an angiograph. Severe left ventricular dysfunction was defined by an ejection fraction of <30% on the first echocardiogram performed during hospitalization, and pulmonary hypertension was indicated by a pulmonary arterial systolic pressure of ≥37 mmHg on the same exam. Lastly, moderate or more severe mitral and tricuspid regurgitation—graded by experienced echocardiographers using the American Society of Echocardiography guidelines—was used to define valvular heart disease [23].

### 2.4. Statistical Analysis

Continuous variables were presented as medians and interquartile ranges (IQRs), or means and standard deviations (SDs), while categorical variables were reported as frequencies and percentages. The comparisons of baseline characteristics between the AKI and non-AKI patients, as well as between the age strata in the whole cohort, were performed using Pearson’s Chi-Squared test for categorical variables and Student’s *t*-test for continuous variables. The inter-layer homogeneity (comparison of the study groups between the age strata) was assessed using the Breslow–Day and two-way analysis of variance (two-way ANOVA) tests for categorial and continuous variables, respectively.

Kaplan–Meier survival curves were used to estimate the probability of mortality following an AKI, and comparisons were made using the log-rank test. Univariable and multivariable Cox regression analyses were conducted for the whole sample and for each age stratum, and the results are reported as hazard ratios (HRs) or adjusted hazard ratios (AdjHRs), along with 95% confidence intervals (CIs). An interaction analysis was undertaken for the whole sample to determine the relative prognostic value of an AKI in relation to age. Lastly, a sub-group analysis was performed, in which the interaction models were applied for the investigated sub-populations (defined by the patients’ sex, type of AMI and ethnicity). The variables with a *p*-value of <0.1 in the univariable analysis were included in the multivariable model. A two-sided *p*-value of <0.05 was considered statistically significant. Statistical analyses were performed using the Statistical Package for the Social Sciences SPSS, version 29 (IBM Corporation, Armonk, NY, USA).

## 3. Results

### 3.1. Study Population and Strata

Between 2002 and 2017, a total of 17,656 patients with an AMI were admitted to the SUMC. Of these, 10,511 patients met this study’s inclusion and exclusion criteria, which consisted of 6132 younger patients (58.3%) and 4379 elderly patients (41.7%) (Supplementary Figure S1). The study population was predominantly male, with a higher percentage of males observed among the younger group. In younger patients, the main risk factors included smoking and a family history of ischemic heart disease (IHD) and a ST elevation myocardial infarction (STEMI) was the most common presentation. More than 90% of these younger patients received invasive treatment. In contrast, the elderly group exhibited a higher percent of women than in the younger stratum, along with a higher prevalence of traditional risk factors, reduced LV function and multivessel disease on angiographs (Supplementary Table S2).

A total of 1602 patients (15.2% of the study cohort) developed an AKI during hospitalization. In the whole cohort, AKIs were more frequently observed in older patients; women; Jews (vs. minorities); and individuals with diabetes, hypertension and peripheral vascular disease. In addition, the incidence of AKIs was higher among the patients with a non-ST elevation myocardial infarction (NSTEMI) than the patients with a STEMI. The echocardiographic findings show that the AKI patients were more likely to have severe LV dysfunction and pulmonary hypertension, while the angiographic results reveal a higher prevalence of multivessel disease. The patients with an AKI also experienced more short-term complications, including cardiogenic shock, gastrointestinal bleeding, and the need for mechanical ventilation and blood transfusions (Supplementary Table S3).

The incidence of AKIs was lower in the younger patients (n = 688, 11.2%) as compared with the elderly patients (n = 914, 20.9%; *p* < 0.001). In the elderly patients, AKIs were more commonly associated with obesity, while in the younger patients, they were more frequently linked to the female gender. In both age groups, AKIs were associated with a higher prevalence of diabetes, hypertension, chronic obstructive pulmonary disease (COPD) and peripheral vascular disease. Additionally, in-hospital complications were more frequent in the patients with an AKI across both age groups (Table 1).

### 3.2. Follow-Up and Outcomes

**Figure 1:**
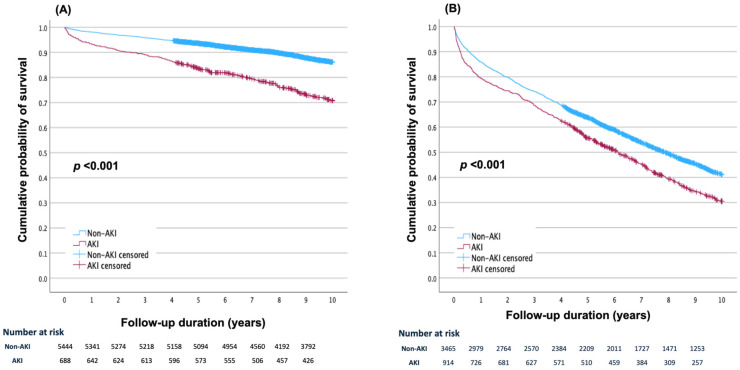
Cumulative survival functions for post-acute myocardial infarction all-cause mortality throughout the follow-up period up to 10 years in the groups of acute kidney injury (AKI) and non-AKI patients by age stratum: (**A**) among younger patients; (**B**) among elderly patients.

The follow-up period extended up to 3652 days (10 years), with a median follow-up of 3542 (IQR: 2236–3652) days. During this period, overall, 3459 patients (32.9%) died: 2566 deaths (58.6%) among the elderly and 893 deaths (14.6%) in the younger stratum. The patients who developed an AKI had a significantly higher mortality rate, with 807 deaths (50.4%) compared with 2664 deaths (29.9%) in patients without an AKI (*p* < 0.001) (Supplementary Figure S2). This increased mortality of the patients with an AKI was evident in both age groups: 27.9% versus 12.9% in the younger group, and 66.0% versus 56.7% in the elderly group (*p* < 0.001 for each). The cumulative survival throughout the follow-up period among the younger patients and among the elderly is presented in Figure 1.

A univariable analysis for the whole cohort revealed that the patients with an AKI had a significantly higher risk of death, with an HR of 1.936 (95% CI: 1.789–2.096, *p* < 0.001) compared with patients without an AKI. Among the younger patients, an AKI was related to a 2.378-fold increased risk of death (95% CI: 2.027–2.789, *p* < 0.001), whereas in the elderly patients, an AKI was associated with a 1.302-fold increased risk of death (95% CI: 1.188–1.426, *p* < 0.001). The impact of an AKI on mortality was significantly greater in younger patients compared with the elderly patients (*p*-for-interaction < 0.001).

### 3.3. Multivariable Analysis

After adjusting for the baseline characteristics, AKIs remained independently associated, with a 1.354-fold increased risk of mortality in the whole study cohort (95% CI: 1.240–1.479, *p* < 0.001). Additional factors associated with increased mortality included older age (≥65 years), diabetes, COPD, malignancy and alcohol abuse. A STEMI was associated with a higher risk for mortality compared with a NSTEMI, and severe LV dysfunction was a significant predictor of death. An invasive treatment, including either percutaneous coronary intervention (PCI) or coronary artery bypass graft surgery (CABG), was associated with lower mortality compared with conservative management. Chronic ischemic heart disease (CIHD) was also found to be associated with better survival (Supplementary Table S4).

Multivariable analysis by age strata demonstrated that AKI was a significant risk factor for long-term mortality in each age stratum. The AdjHRs were 1.634 (95% CI: 1.363–1.959, *p* < 0.001) for the younger patients and 1.278 (95% CI: 1.154–1.415, *p* < 0.001) for the elderly patients, with the association being stronger in the younger cohort (*p*-for-interaction = 0.020). Other factors, such as cardiomegaly, diabetes, COPD, neurological disorders, malignancy, alcohol abuse and mitral regurgitation, had more pronounced associations with mortality in the younger patients (Table 2).

### 3.4. Sub-Group Analysis

A sub-group analysis revealed that an AKI was a significant predictor of long-term mortality across all the age groups in every sub-population examined—including women, men, NSTEMI and STEMI patients, and patients that underwent a PCI or CABG. Moreover, the strength of the association between an AKI and mortality varied significantly across the age strata in both women and men, among the NSTEMI (but not STEMI) patients and among the different invasive treatment strategies. Notably, the adverse impact of an AKI on mortality was particularly pronounced in the younger population and persisted from one year up to ten years of follow-up (Table 3).

## 4. Discussion

This study examined the outcomes of patients with an acute myocardial infarction (AMI), with a specific focus on the incidence of acute kidney injuries (AKIs) across two distinct age groups and its association with long-term mortality. Among the 10,511 eligible patients included in the analysis, AKIs were observed in 15.2% of cases, with a significantly higher incidence among the elderly patients compared with their younger counterparts (20.9% vs. 11.2%). The presence of an AKI significantly increased the risk of death in both age strata, but the association was notably stronger in the younger patients (AdjHR: 1.634, 95% CI: 1.363–1.959, *p* < 0.001) than in the elderly patients (AdjHR: 1.278, 95% CI: 1.154–1.415, *p* < 0.001), as evidenced by a significant interaction (*p*-for-interaction = 0.020). Mortality was also influenced by multiple comorbidities, including diabetes, COPD, malignancy and severe LV dysfunction. Importantly, the patients who underwent invasive treatment strategies demonstrated better survival outcomes compared with those managed conservatively (after adjusting for baseline characteristics), highlighting the potential benefits of aggressive therapeutic approaches across both age groups.

The mechanism underlying an AKI after an AMI, which is categorized as cardiorenal syndrome type 1, is multifaceted and involves several interrelated processes. These include activation of the renin–angiotensin system, dysregulation of the nitric oxide (NO) pathway and the release of inflammatory mediators, which collectively contribute to direct tubular damage. Additionally, exposure to contrast agents during diagnostic or therapeutic procedures can exacerbate kidney injury. These mechanisms are further compounded by rapid hemodynamic changes, particularly in the setting of coronary artery occlusion, which leads to impaired left ventricular (LV) function, reduced stroke volume and altered renal perfusion [1,4,5,6,24,25,26,27]. Notably, the protective effect of invasive treatment strategies may indicate that contrast-induced nephropathy played a relatively minor role in the overall kidney injury. Although these findings are shaped by potential confounding factors and differences in patient characteristics, they are consistent with previous research suggesting that underlying risk factors and the severity of cardiac injury are the primary drivers of AKI development [6,9,28].

The strong association between a post-AMI AKI and mortality, particularly in the younger patients, highlights the kidneys’ sensitivity to injury and their potential role as early markers of comorbidity and prognosis. This association remained significant after adjusting for multiple risk factors, suggesting that some comorbidities might be partially concealed or underestimated. The significant influence of an AKI on long-term mortality in patients with an AMI is well documented [7,8,9,10,11,12]. Advanced age is recognized as an independent risk factor for both MACEs and AKIs [13,14,15,16,17]. However, a recent study suggests that the prognostic impact of an AKI diminishes progressively with increasing age, whereas in younger patients, an AKI serves as a significant warning sign, indicating an increased risk of mortality [29]. Our findings highlight the critical importance of an AKI in younger individuals, revealing its strong association, with nearly a twofold increase in mortality. This underscores the urgency of early detection and proactive management of kidney injury in this population, where timely intervention could significantly improve outcomes and mitigate long-term risks. These insights are particularly valuable for identifying high-risk younger patients who may be more susceptible to complications. By pinpointing this vulnerable subgroup, clinicians can prioritize aggressive risk factor control and implement tailored strategies to prevent the progression of an AKI and its associated consequences. The use of novel therapeutic agents that provide direct protection to both the heart and kidneys should be considered. This includes sodium–glucose cotransporter 2 inhibitors (SGLT2Is) [30,31], glucagon-like peptide receptor agonists (GLP1RAs) [32,33] and nonsteroidal mineralocorticoid receptor antagonists (MRAs) [34,35]. Moreover, this approach holds particular relevance for younger patients, who often have longer life expectancies and, therefore, stand to gain the most from interventions aimed at preserving kidney function and improving long-term outcomes. This study also highlights the importance of comprehensive management of comorbidities, such as diabetes, COPD and LV dysfunction, which significantly contributed to the mortality risk.

Several limitations should be considered when interpreting these findings. First, the retrospective design of this study introduced an inherent risk of residual confounding, despite our adjustments for the baseline characteristics. The lack of detailed data on contrast agent volumes and comprehensive laboratory results may have contributed to an inaccurate assessment of the relationship between an AKI and mortality. Second, the kidney function assessment was based solely on serum creatinine measurements, without incorporating urine albumin levels. This limitation may have resulted in a misclassification of CKD at the baseline, thereby potentially diluting the observed associations between renal dysfunction and long-term outcomes. Third, the exclusion of patients with missing data might have introduced a selection bias, as those with incomplete records could systematically differ from those included, thus affecting the representativeness and generalizability of the study population. Fourth, the lack of detailed information on the causes of mortality restricted our ability to ascertain the specific mechanisms through which an AKI contributes to an increased risk of death. Finally, as this study was conducted at a single medical center, the findings may not fully reflect patient populations or clinical practices in other settings. Despite these limitations, the large cohort size and consistency with previous research lend credibility to our conclusions.

## 5. Conclusions

This study suggests an association between an AKI following an AMI and long-term mortality, particularly in younger patients. However, external validation is needed, and further research is required to assess the benefits of early intervention and follow-up.

## Figures and Tables

**Table 1:** Baseline characteristics of the study population: comparison between the acute kidney injury (AKI) and non-AKI groups in each age stratum (younger and elderly), and inter-layer homogeneity between the strata.

Variable	Age < 65 Years			Age ≥ 65 Years			Homogeneity *p*
	n = 6132			n = 4379			
	Non-AKI	AKI	*p*	Non-AKI	AKI	*p*	
	n = 5444	n = 688		n = 3465	n = 914		
**Demographics**							
Age, years—mean (SD)	52.48 (7.723)	54.77 (6.906)	<0.001	75.03 (7.31)	75.06 (7.00)	0.915	<0.001
Sex, males	4680 (86.0)	564 (82.0)	0.005	2185 (63.1)	569 (62.3)	0.654	0.045
Ethnicity, minorities	1363 (25.0)	146 (21.2)	0.029	405 (11.7)	105 (11.5)	0.867	0.199
**Cardiac diseases**							
Cardiomegaly	240 (4.4)	67 (9.7)	<0.001	330 (9.5)	127 (13.9)	<0.001	0.02
Supraventricular arrhythmias	253 (4.6)	59 (8.6)	<0.001	708 (20.4)	250 (27.4)	<0.001	0.115
CHF	343 (6.3)	128 (18.6)	<0.001	582 (16.8)	284 (31.1)	<0.001	0.003
Pulmonary heart disease	121 (2.2)	29 (4.2)	0.001	370 (10.7)	128 (14.0)	0.005	0.138
s/p MI	476 (8.7)	141 (20.5)	<0.001	471 (13.6)	217 (23.7)	<0.001	0.028
CIHD	4819 (88.5)	639 (92.9)	0.001	2661 (76.8)	789 (86.3)	<0.001	0.519
s/p PCI	594 (10.9)	154 (22.4)	<0.001	477 (13.8)	177 (19.4)	<0.001	0.001
s/p CABG	183 (3.4)	49 (7.1)	<0.001	346 (10.0)	81 (8.9)	0.308	<0.001
AV block	107 (2.0)	24 (3.5)	0.009	168 (4.8)	40 (4.4)	0.551	0.016
**Cardiovascular risk factors**							
Diabetes mellitus	1627 (29.9)	314 (45.6)	<0.001	1423 (41.1)	474 (51.9)	<0.001	0.029
Dyslipidemia	4679 (85.9)	593 (86.2)	0.862	2755 (79.5)	740 (81.0)	0.33	0.635
Hypertension	2206 (40.5)	368 (53.5)	<0.001	2409 (69.5)	688 (75.3)	0.001	0.046
Obesity	1452 (26.7)	204 (29.7)	0.097	605 (17.5)	193 (21.1)	0.011	0.493
Smoking	3727 (68.5)	434 (63.1)	0.004	984 (28.4)	248 (27.1)	0.449	0.137
PVD	294 (5.4)	82 (11.9)	<0.001	393 (11.3)	185 (20.2)	<0.001	0.278
Family history of IHD	1030 (18.9)	111 (16.1)	0.077	88 (2.5)	32 (3.5)	0.113	0.26
**Other disorders**							
COPD	193 (3.5)	47 (6.8)	<0.001	389 (11.2)	123 (13.5)	0.062	0.015
Neurological disorders	327 (6.0)	93 (13.5)	<0.001	694 (20.0)	212 (23.2)	0.036	<0.001
Malignancy	69 (1.3)	13 (1.9)	0.181	183 (5.3)	66 (7.2)	0.024	0.832
Anemia	1362 (25.0)	416 (60.5)	<0.001	1590 (45.9)	611 (66.8)	<0.001	<0.001
Schizophrenia/psychosis	58 (1.1)	17 (2.5)	0.002	55 (1.6)	26 (2.8)	0.012	0.48
Alcohol/drug addiction	181 (3.3)	27 (3.9)	0.413	52 (1.5)	12 (1.3)	0.674	0.423
History of malignancy	103 (1.9)	21 (3.1)	0.042	261 (7.5)	63 (6.9)	0.511	0.037
**Administrative characteristics of the hospitalization**							
LOS, >7 days	1950 (35.8)	568 (82.6)	<0.001	1762 (50.9)	804 (88.0)	<0.001	0.221
STEMI	3524 (64.7)	385 (56.0)	<0.001	1401 (40.4)	366 (40.0)	0.831	0.002
**Results of echocardiography**							
Echocardiography performance	4888 (89.8)	578 (84.0)	<0.001	2560 (73.9)	696 (76.1)	0.163	<0.001
Severe LV dysfunction	323 (6.6)	109 (18.9)	<0.001	284 (11.1)	123 (17.7)	<0.001	<0.001
LV hypertrophy	141 (2.9)	23 (4.0)	0.145	163 (6.4)	34 (4.9)	0.146	0.039
Mitral regurgitation	79 (1.6)	25 (4.3)	<0.001	172 (6.7)	57 (8.2)	0.178	0.004
Tricuspid regurgitation	27 (0.6)	10 (1.7)	0.001	109 (4.3)	40 (5.7)	0.095	0.041
Pulmonary hypertension	67 (1.4)	26 (4.5)	<0.001	230 (9.0)	89 (12.8)	0.003	0.002
**Results of angiography**							
Angiography performance	4722 (86.7)	559 (81.3)	<0.001	2252 (65.0)	603 (66.0)	0.58	0.001
Measure of CAD, none or non-significant	170 (3.6)	13 (2.3)	<0.001	121 (5.4)	10 (1.7)	<0.001	
One vessel	1699 (36.0)	94 (16.8)		504 (22.4)	70 (11.6)		
Two vessels	1476 (31.3)	114 (20.4)		637 (28.3)	110 (18.2)		
Three vessels/LM	1377 (29.2)	338 (60.5)		990 (44.0)	413 (68.5)		
**Type of treatment**							
Noninvasive	404 (7.4)	39 (5.7)	<0.001	1049 (30.3)	208 (22.8)	<0.001	
PCI	4373 (80.3)	270 (39.2)		2107 (60.8)	289 (31.6)		
CABG	667 (12.3)	379 (55.1)		309 (8.9)	417 (45.6)		
**eGFR < 90 mL/min/1.73 m^2^**	2341 (43.0)	345 (50.1)	<0.001	2506 (72.3)	666 (72.9)	0.744	0.025
**In-hospital course**							
Cardiac arrest	12 (0.2)	7 (1.0)	<0.001	5 (0.1)	11 (1.2)	<0.001	0.403
Cardiogenic shock	43 (0.8)	26 (3.8)	<0.001	33 (1.0)	35 (3.8)	<0.001	0.617
Intra-aortic balloon pulsation	97 (1.8)	63 (9.2)	<0.001	54 (1.6)	73 (8.0)	<0.001	0.957
Any form of pacing	45 (0.8)	21 (3.1)	<0.001	68 (2.0)	28 (3.1)	0.043	0.011
Mechanical ventilation	85 (1.6)	79 (11.5)	<0.001	74 (2.1)	118 (12.9)	<0.001	0.401
Gastrointestinal bleeding	56 (1.0)	21 (3.1)	<0.001	59 (1.7)	45 (4.9)	<0.001	0.968
Blood transfusion	291 (5.3)	242 (32.5)	<0.001	329 (9.5)	375 (41.0)	<0.001	0.005
Sepsis	6 (0.1)	10 (1.5)	<0.001	17 (0.5)	44 (4.8)	<0.001	0.652

Data presented as the number of patients and percent of categories for all investigated variables except age. AKI, acute kidney injury; SD, standard deviation; CHF, congestive heart failure; s/p, status post; MI, myocardial infarction; CIHD, chronic ischemic heart disease; PCI, percutaneous coronary intervention; CABG, coronary artery bypass graft; AV, atrioventricular; PVD, peripheral vascular disease; IHD, ischemic heart disease; COPD, chronic obstructive pulmonary disease; LOS, length of stay; STEMI, ST elevation myocardial infarction; LV, left ventricular; CAD, coronary artery disease; LM, left main; eGFR, estimated glomerular filtration rate.

**Table 2:** Adjusted relative risk (adjusted hazard ratio) for long-term (up to 10 years of follow-up) post-acute myocardial infarction all-cause mortality by age stratum (younger and elderly), and disparities between the strata—multivariable analysis.

Variable	Age < 65 Years			Age ≥ 65 Years			*p*-for-Interaction
	AdjHR	(95% CI)	*p*	AdjHR	(95% CI)	*p*	
AKI (yes vs. no)	1.634	(1.363–1.959)	<0.001	1.278	(1.154–1.415)	<0.001	0.020
Age, years (1-year increase)	1.043	(1.032–1.054)	<0.001	1.053	(1.047–1.059)	<0.001	0.128
Ethnicity (minorities vs. others)	1.314	(1.125–1.535)	<0.001	1.095	(0.965–1.243)	0.160	0.075
Cardiomegaly	1.549	(1.241–1.932)	<0.001	1.082	(0.957–1.224)	0.210	0.005
Supraventricular arrhythmias	1.413	(1.141–1.749)	0.002	1.274	(1.165–1.393)	<0.001	0.382
CHF	1.304	(1.071–1.588)	0.008	1.291	(1.174–1.420)	<0.001	0.911
Pulmonary heart disease	1.074	(0.787–1.466)	0.651	1.236	(1.088–1.405)	0.001	0.404
CIHD	0.943	(0.748–1.188)	0.617	0.891	(0.797–0.996)	0.042	0.659
s/p MI	1.298	(1.081–1.558)	0.005	1.215	(1.094–1.349)	<0.001	0.544
Diabetes mellitus	1.712	(1.483–1.977)	<0.001	1.271	(1.172–1.378)	<0.001	<0.001
Dyslipidemia	0.764	(0.641–0.910)	0.003	0.892	(0.811–0.981)	0.018	0.125
Smoking	1.129	(0.975–1.309)	0.106	1.138	(1.029–1.257)	0.012	0.943
PVD	1.607	(1.320–1.956)	<0.001	1.437	(1.291–1.600)	<0.001	0.317
COPD	2.320	(1.874–2.872)	<0.001	1.691	(1.509–1.896)	<0.001	0.009
Neurological disorders	1.945	(1.620–2.336)	<0.001	1.437	(1.314–1.572)	<0.001	0.003
Malignancy	3.265	(2.318–4.598)	<0.001	1.872	(1.621–2.163)	<0.001	0.003
Anemia	1.398	(1.204–1.622)	<0.001	1.315	(1.212–1.428)	<0.001	0.478
Schizophrenia/psychosis	1.783	(1.214–2.619)	0.003	2.058	(1.621–2.612)	<0.001	0.555
Alcohol/drug addiction	3.470	(2.736–4.401)	<0.001	1.873	(1.392–2.521)	<0.001	0.001
Type of AMI (NSTEMI vs. STEMI)	0.823	(0.715–0.948)	0.007	0.858	(0.788–0.936)	<0.001	0.621
Severe LV dysfunction	1.681	(1.373–2.056)	<0.001	1.569	(1.378–1.787)	<0.001	0.558
LV hypertrophy	1.170	(0.840–1.628)	0.353	1.312	(1.091–1.578)	0.004	0.54
Mitral regurgitation	2.109	(1.556–2.859)	<0.001	1.232	(1.042–1.457)	0.015	0.002
Pulmonary hypertension	1.121	(0.785–1.601)	0.528	1.204	(1.027–1.411)	0.022	0.736
Type of treatment:							
Noninvasive	1 (ref.)			1 (ref.)			
PCI	0.549	(0.436–0.692)	<0.001	0.524	(0.468–0.586)	<0.001	0.740
CABG	0.330	(0.248–0.441)	<0.001	0.341	(0.291–0.399)	<0.001	0.820

AdjHR, adjusted hazard ratio; CI, confidence interval; AKI, acute kidney injury; CHF, congestive heart failure; CIHD, chronic ischemic heart disease; s/p, status post; MI, myocardial infarction; PVD, peripheral vascular disease; COPD, chronic obstructive pulmonary disease; NSTEMI, non-ST elevation myocardial infarction; STEMI, ST elevation myocardial infarction; LV, left ventricular; PCI, percutaneous coronary intervention; CABG, coronary artery bypass graft; ref., reference group (category).

**Table 3:** Relationship between an acute kidney injury and the risk for short (one-year follow-up) and long-term (up to 10 years of follow-up) post-acute myocardial infarction all-cause mortality by age stratum (younger and elderly), and disparities between the strata for the investigated sub-populations—sub-group analysis.

Sub-Population *	Age < 65 Years			Age ≥ 65 Years			*p*-for-Interaction
	AdjHR	(95% CI)	*p*	AdjHR	(95% CI)	*p*	
Women	2.167	(1.437–3.268)	<0.001	1.337	(1.138–1.570)	<0.001	0.001
Men	1.489	(1.211–1.831)	<0.001	1.242	(1.087–1.419)	0.001	0.005
NSTEMI	1.673	(1.287–2.174)	<0.001	1.227	(1.080–1.393)	0.002	<0.001
STEMI	1.552	(1.197–2.013)	<0.001	1.446	(1.212–1.724)	<0.001	0.178
Non-invasive	1.506	(0.920–2.466)	0.103	1.298	(1.104–1.525)	0.002	0.084
PCI	1.618	(1.258–2.082)	<0.001	1.217	(1.034–1.434)	0.019	0.003
CABG	1.615	(1.149–2.270)	0.006	1.423	(1.108–1.827)	0.006	0.401
One-year follow-up	2.310	(1.561–3.419)	<0.001	1.660	(1.385–1.991)	<0.001	0.046
Two–ten-year follow-up **	1.499	(1.220–1.842)	<0.001	1.129	(0.999–1.277)	0.053	0.002

* Each line in this table represents the results of a separate interactive multivariable model; adjusted for cardiomegaly, supraventricular arrhythmias, congestive heart failure, pulmonary heart disease, chronic ischemic heart disease, history of myocardial infarction, diabetes mellitus, dyslipidemia, smoking, peripheral vascular disease, chronic obstructive pulmonary disease, neurological disorders, malignancy, anemia, schizophrenia/psychosis, alcohol/drug addiction, left ventricular dysfunction, left ventricular hypertrophy, mitral regurgitation, pulmonary hypertension and type of treatment for acute myocardial infarction. ** For the persons who survived the first year of the follow-up (landmark analysis). AdjHR, adjusted hazard ratio; CI, confidence interval; STEMI, ST elevation myocardial infarction; NSTEMI, non-ST elevation myocardial infarction; PCI, percutaneous coronary intervention; CABG, coronary artery bypass graft.

## Data Availability

The data underlying this article will be shared upon reasonable request to the corresponding author.

## Supplementary Materials

The following supporting information can be downloaded from https://www.mdpi.com/article/10.3390/jcdd12040133/s1: Figure S1. Study flow chart; Table S1. Diagnoses and interventions according to the International Classification of Diseases, Ninth Revision, Clinical Modification (ICD-9-CM) codes; Table S2. Baseline characteristics of the study population by age strata; Table S3. Baseline characteristics of the study population by AKI group in the whole cohort; Figure S2. Cumulative survival functions throughout the follow-up period in the study cohort by acute kidney injury (AKI) group; Table S4. Mortality risk according to the investigated variables in the whole cohort—multivariable analysis.
