# Supplementary material for: Age-Dependent Risk of Long-Term All-Cause Mortality in Patients Post-Myocardial Infarction and Acute Kidney Injury
Source: J Cardiovasc Dev Dis. 2025 Apr 3;12(4):133. doi: 10.3390/jcdd12040133 (PMC12028031; doi:10.3390/jcdd12040133)
Supplement: Supplementary file 1 [file jcdd-12-00133-s001.zip › jcdd-3478409-supplementary.pdf]

Supplementary Figure S1. Study flow chart

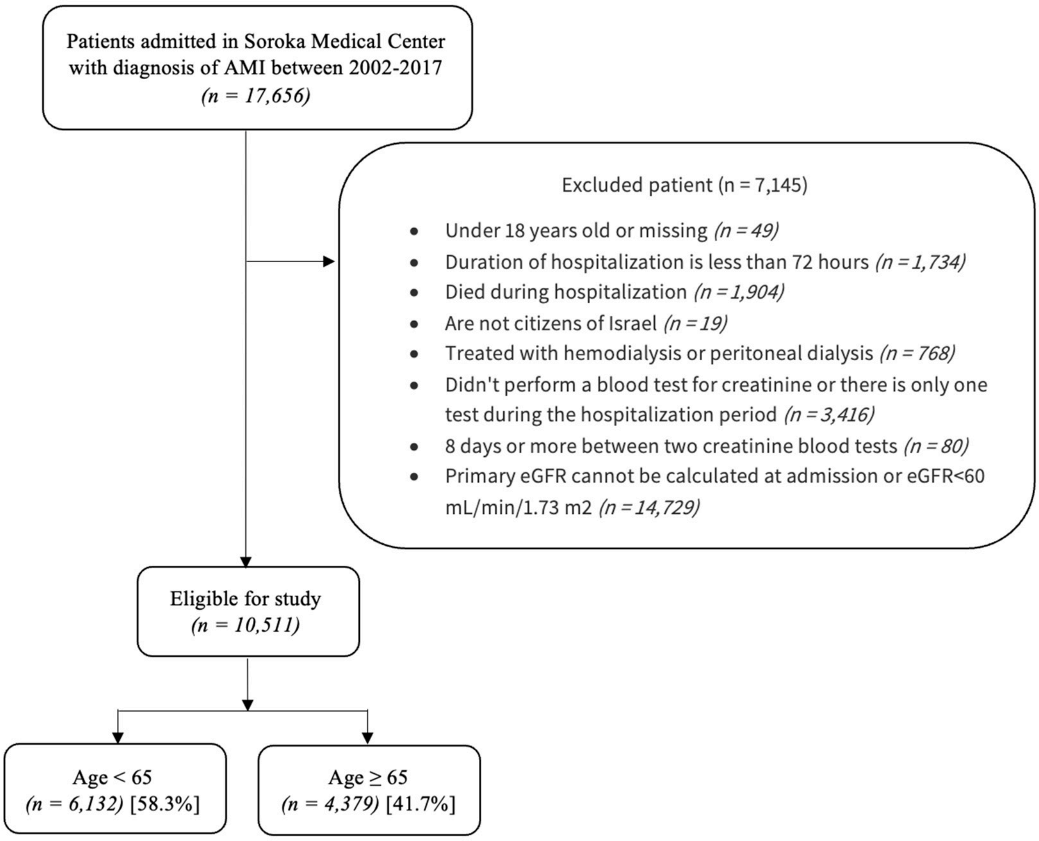

Supplementary Table S1. Diagnoses and interventions according to the International Classification of Diseases, Ninth Revision, Clinical Modification (ICD-9-CM) codes. * - All codes in this group.

Diagnosis description	Diagnosis codes
Cardiomegaly	429.3
Atrial Fibrillation / Flutter	427.3
Congestive heart failure	428*
Chronic pulmonary heart disease	416*
History of myocardial infarction	412
Atrioventricular block	426.0, 426.1*
Smoking	305.1
Peripheral vascular disease	443*, 444.2*
Hypertension	401*
Dyslipidemia	272.0-272.4
Chronic obstructive pulmonary disease	490*-496*
Neurological disorders	340-342*, 344*, 433*, 434*, 437*, 438*
Malignancy	148–208*, 230–233*
ST elevation myocardial infarction	410.0*-410.6*
Non-ST elevation myocardial infarction	410.7*-410.9*
Percutaneous coronary intervention	36.06-36.09
Coronary artery bypass surgery	36.2-26.9*

Supplementary Table S2. Baseline characteristics of the study population by age strata

Variable	Total cohort<br>n=10511	Age < 65 years<br>n = 6132	Age ≥ 65 years<br>n = 4379	p
**Demographics**				
Age, years - Mean (SD)	62.03 (13.30)	52.74; 7.67	75.03; 7.25	<0.001
Sex, Males	7998 (76.1)	5244 (85.5)	2754 (62.9)	<0.001
Ethnicity, Minorities	2019 (19.2)	1509 (24.6)	510 (11.6)	<0.001
**Cardiac diseases**				
Cardiomegaly	764 (7.3)	307 (5)	457 (10.4)	<0.001
Supraventricular arrhythmias	1270 (12.1)	312 (5.1)	958 (21.9)	<0.001
CHF	1337 (12.7)	471 (7.7)	866 (19.8)	<0.001
Pulmonary heart disease	648 (6.2)	150 (2.4)	498 (11.4)	<0.001
s/p MI	1305 (12.4)	617 (10.1)	688 (15.7)	<0.001
CIHD	8908 (84.7)	5458 (89)	3450 (78.8)	<0.001
s/p PCI	1402 (13.3)	748 (12.2)	654 (14.9)	<0.001
s/p CABG	659 (6.3)	232 (3.8)	427 (9.8)	<0.001
AV block	339 (3.2)	131 (2.1)	208 (4.7)	<0.001
**Cardiovascular risk factors**				
Diabetes mellitus	3838 (36.5)	1941 (31.7)	1897 (43.3)	<0.001
Dyslipidemia	8767 (83.4)	5272 (86)	3495 (79.8)	<0.001
Hypertension	5671 (54)	2574 (42)	3097 (70.7)	<0.001
Obesity	2454 (23.3)	1656 (27)	798 (18.2)	<0.001
Smoking	5393 (51.3)	4161 (67.9)	1232 (28.1)	<0.001
PVD	954 (9.1)	376 (6.1)	578 (13.2)	<0.001
Family history of IHD	1261 (12.0)	1141 (18.6)	120 (2.7)	<0.001
**Other disorders**				
COPD	752 (7.2)	240 (3.9)	512 (11.7)	<0.001
Neurological disorders	1326 (12.6)	420 (6.8)	906 (20.7)	<0.001
Malignancy	331 (3.1)	82 (1.3)	249 (5.7)	<0.001
Anemia	3979 (37.9)	1778 (29.0)	2201 (50.3)	<0.001
Schizophrenia/Psychosis	156 (1.5)	75 (1.2)	81 (1.8)	0.009
Alcohol/drug addiction	272 (2.6)	208 (3.4)	64 (1.5)	<0.001
History of malignancy	448 (4.3)	124 (2.0)	324 (7.4)	<0.001
**Administrative characteristics of the hospitalization**				
LOS, >7 days	5084 (48.4)	2518 (41.1)	2566 (58.6)	<0.001
STEMI	5676 (54.0)	3909 (63.7)	1767 (40.4)	<0.001
**Results of echocardiography**				
Echocardiography performance	8722 (83.0)	5466 (89.1)	3256 (74.4)	<0.001
Severe LV dysfunction	839 (9.6)	432 (7.9)	407 (12.5)	<0.001
LV hypertrophy	361 (4.1)	164 (3)	197 (6.1)	<0.001

Mitral regurgitation	333 (3.8)	104 (1.9)	229 (7.0)	<0.001
Tricuspid regurgitation	186 (2.1)	37 (0.7)	149 (4.6)	<0.001
Pulmonary hypertension	412 (4.7)	93 (1.7)	319 (9.8)	<0.001
**Results of angiography**				
Angiography performance	8136 (77.4)	5281 (86.1)	2855 (65.2)	<0.001
Measure of CAD, No or non-significant	314 (3.9)	183 (3.5)	131 (4.6)	<0.001
One vessel	2367 (29.1)	1793 (34)	574 (20.1)	
Two vessels	2337 (28.7)	1590 (30.1)	747 (26.2)	
Three vessels/ LM	3118 (38.3)	1715 (32.5)	1403 (49.1)	
**Type of treatment**				
Noninvasive	1700 (16.2)	443 (7.2)	1257 (28.7)	<0.001
PCI	7039 (67.0)	4643 (75.7)	2396 (54.7)	
CABG	1772 (16.9)	1046 (17.1)	726 (16.6)	
eGFR<90mL/min/1.73 m^2	5858 (55.7)	2686 (43.8)	3172 (72.4)	<0.001
**In-hospital course**				
Cardiac arrest	35 (0.3)	19 (0.3)	16 (0.4)	0.626
Cardiogenic shock	137 (1.3)	69 (1.1)	68 (1.6)	0.057
Intra-aortic balloon pump	287 (2.7)	160 (2.6)	127 (2.9)	0.367
Any form of pacing	162 (1.5)	66 (1.1)	96 (2.2)	<0.001
Mechanical ventilation	356 (3.4)	164 (2.7)	192 (4.4)	<0.001
Gastrointestinal bleeding	181 (1.7)	77 (1.3)	104 (2.4)	<0.001
Blood transfusion	1237 (11.8)	533 (8.7)	704 (16.1)	<0.001
Sepsis	77 (0.7)	16 (0.3)	61 (1.4)	<0.001

Data presented as the number of patients and percent of categories for all investigated variables except age. SD, standard deviation; CHF, congestive heart failure; s/p, status post; MI, myocardial infarction; CIHD, chronic ischemic heart disease; PCI, percutaneous coronary intervention; CABG, coronary artery bypass graft; AV, atrioventricular; PVD, peripheral vascular disease; IHD, ischemic heart disease; COPD, chronic obstructive pulmonary disease; LOS, length of stay; STEMI, ST-elevation myocardial infarction; LV, left ventricular; CAD, coronary artery disease; LM, left main; eGFR, estimated glomerular filtration rate.

Supplementary Table S3. Baseline characteristics of the study population by AKI groups in the whole cohort

Variable	Total cohort n=10511	non-AKI n=8909	AKI n=1602	p
**Demographics**				
Age, years - Mean (SD)	62.03 (13.30)	61.25 (13.34)	66.35 (12.21)	<0.001
Sex, Males	7998 (76.1)	6865 (77.1)	1133 (70.7)	<0.001
Ethnicity, Minorities	2019 (19.2)	1768 (19.8)	251 (15.7)	<0.001
**Cardiac diseases**				
Cardiomegaly	764 (7.3)	570 (6.4)	194 (12.1)	<0.001
Supraventricular arrhythmias	1270 (12.1)	961 (10.8)	309 (19.3)	<0.001
CHF	1337 (12.7)	925 (10.4)	412 (25.7)	<0.001
Pulmonary heart disease	648 (6.2)	491 (5.5)	157 (9.8)	<0.001
s/p MI	1305 (12.4)	947 (10.6)	358 (22.3)	<0.001
CIHD	8908 (84.7)	7480 (84.0)	1428 (89.1)	<0.001
s/p PCI	1402 (13.3)	1071 (12.0)	331 (20.7)	<0.001
s/p CABG	659 (6.3)	529 (5.9)	130 (8.1)	0.001
AV block	339 (3.2)	275 (3.1)	64 (4.0)	0.058
**Cardiovascular risk factors**				
Diabetes mellitus	3838 (36.5)	3050 (34.2)	788 (49.2)	<0.001
Dyslipidemia	8767 (83.4)	7434 (83.4)	1333 (83.2)	0.816
Hypertension	5671 (54.0)	4615 (51.8)	1056 (65.9)	<0.001
Obesity	2454 (23.3)	2057 (23.1)	397 (24.8)	0.14
Smoking	5393 (51.3)	4711 (52.9)	682 (42.6)	<0.001
PVD	954 (9.1)	687 (7.7)	267 (16.7)	<0.001
Family history of IHD	1261 (12)	1118 (12.5)	143 (8.9)	<0.001
**Other disorders**				
COPD	752 (7.2)	582 (6.5)	170 (10.6)	<0.001
Neurological disorders	1326 (12.6)	1021 (11.5)	305 (19.0)	<0.001
Malignancy	331 (3.1)	252 (2.8)	79 (4.9)	<0.001
Anemia	3979 (37.9)	2952 (33.1)	1027 (64.1)	<0.001
Schizophrenia/Psychosis	156 (1.5)	113 (1.3)	43 (2.7)	<0.001
Alcohol/drug addiction	272 (2.6)	233 (2.6)	39 (2.4)	0.675
History of malignancy	448 (4.3)	364 (4.1)	84 (5.2)	0.035
**Administrative characteristics of the hospitalization**				
LOS, >7 days	5084 (48.4)	3712 (41.7)	1372 (85.6)	<0.001
STEMI	5676 (54.0)	4925 (55.3)	751 (46.9)	<0.001
**Results of echocardiography**				
Echocardiography performance	8722 (83.0)	7448 (83.6)	1274 (79.5)	<0.001
Severe LV dysfunction	839 (9.6)	607 (8.1)	232 (18.2)	<0.001

LV hypertrophy	361 (4.1)	304 (4.1)	57 (4.5)	0.516
Mitral regurgitation	333 (3.8)	251 (3.4)	82 (6.4)	<0.001
Tricuspid regurgitation	186 (2.1)	136 (1.8)	50 (3.9)	<0.001
Pulmonary hypertension	412 (4.7)	297 (4.0)	115 (9.0)	<0.001
**Results of angiography**				
Angiography performance	8136 (77.4)	6974 (78.3)	1162 (72.5)	<0.001
Measure of CAD, No or non-significant	314 (3.9)	291 (4.2)	23 (2.0)	<0.001
One vessel	2367 (29.1)	2203 (31.6)	164 (14.1)	
Two vessels	2337 (28.7)	2113 (30.3)	224 (19.3)	
Three vessels/ LM	3118 (38.3)	2367 (33.9)	751 (64.6)	
**Type of treatment**				
Noninvasive	1700 (16.2)	1453 (16.3)	247 (15.4)	<0.001
PCI	7039 (67.0)	6480 (72.7)	559 (34.9)	<0.001
CABG	1772 (16.9)	976 (11.0)	796 (49.7)	
eGFR<90mL/min/1.73 m^2	5858 (55.7)	4847 (54.4)	1011 (63.1)	<0.001
**In-hospital course**				
Cardiac arrest	35 (0.3)	17 (0.2)	18 (1.1)	<0.001
Cardiogenic shock	137 (1.3)	76 (0.9)	61 (3.8)	<0.001
Intra-aortic balloon pump	287 (2.7)	151 (1.7)	136 (8.5)	<0.001
Any form of pacing	162 (1.5)	113 (1.3)	49 (3.1)	<0.001
Mechanical ventilation	236 (3.4)	159 (1.8)	197 (12.3)	<0.001
Gastrointestinal bleeding	181 (1.7)	115 (1.3)	66 (4.1)	<0.001
Blood transfusion	1237 (11.8)	620 (7.0)	617 (38.5)	<0.001
Sepsis	77 (0.7)	23 (0.3)	54 (3.4)	<0.001

Data presented as the number of patients and percent of categories for all investigated variables except age.

AKI, acute kidney injury; SD, standard deviation; CHF, congestive heart failure; s/p, status post; MI, myocardial infarction; CIHD, chronic ischemic heart disease; PCI, percutaneous coronary intervention; CABG, coronary artery bypass graft; AV, atrioventricular; PVD, peripheral vascular disease; IHD, ischemic heart disease; COPD, chronic obstructive pulmonary disease; LOS, length of stay; STEMI, ST-elevation myocardial infarction; LV, left ventricular; CAD, coronary artery disease; LM, left main; eGFR, estimated glomerular filtration rate.

Supplementary Figure S2. Cumulative survival functions throughout the follow-up period in the study cohort by acute kidney injury (AKI) groups

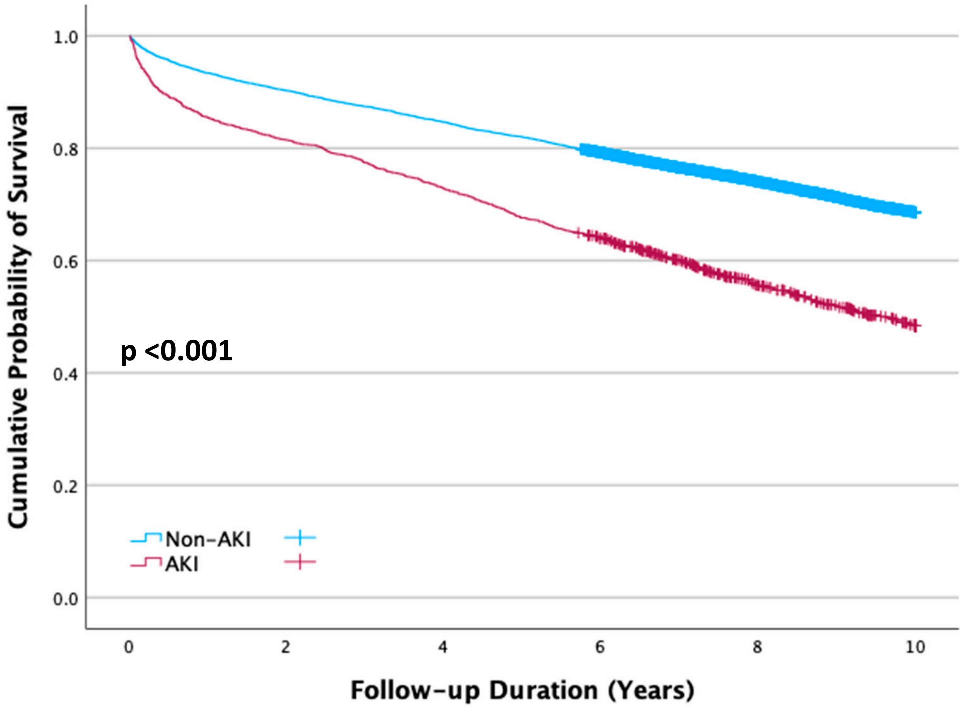

Supplementary Table S4. Mortality risk according to the investigated variables in the whole cohort - multivariable analysis

Variable	B (SE)	AdjHR	(95% CI)	p
Age groups (≥65 vs. <65 years)	0.219 (0.064)	1.245	(1.098 ; 1.412)	<0.001
AKI (Yes vs. No)	0.303 (0.045)	1.354	(1.240 ; 1.479)	<0.001
Age, years (1-year increase)	0.051 (0.003)	1.053	(1.047 ; 1.058)	<0.001
Ethnicity (Minorities vs. Others)	0.168 (0.049)	1.183	(1.073 ; 1.303)	<0.001
Cardiomegaly	0.162 (0.055)	1.176	(1.057 ; 1.308)	0.003
Supraventricular arrhythmias	0.259 (0.042)	1.296	(1.193 ; 1.408)	<0.001
CHF	0.260 (0.043)	1.296	(1.191 ; 1.412)	<0.001
Pulmonary heart disease	0.194 (0.060)	1.214	(1.078 ; 1.367)	0.001
CIHD	-0.122 (0.051)	0.885	(0.801 ; 0.977)	0.016
s/p MI	0.215 (0.046)	1.240	(1.134 ; 1.357)	<0.001
Diabetes mellitus	0.322 (0.035)	1.380	(1.287 ; 1.479)	<0.001
Dyslipidemia	-0.149 (0.042)	0.861	(0.793 ; 0.936)	<0.001
Smoking	0.103 (0.042)	1.108	(1.021 ; 1.203)	0.014
PVD	0.405 (0.048)	1.500	(1.366 ; 1.647)	<0.001
COPD	0.591 (0.051)	1.806	(1.634 ; 1.996)	<0.001
Neurological disorders	0.424 (0.041)	1.528	(1.409 ; 1.656)	<0.001
Malignancy	0.698 (0.068)	2.010	(1.760 ; 2.295)	<0.001
Anemia	0.300 (0.037)	1.350	(1.256 ; 1.45)	<0.001
Schizophrenia/Psychosis	0.731 (0.102)	2.077	(1.700 ; 2.537)	<0.001
Alcohol/drug addiction	0.976 (0.092)	2.653	(2.217 ; 3.176)	<0.001
Type of AMI (NSTEMI vs. STEMI)	-0.170 (0.038)	0.844	(0.784 ; 0.908)	<0.001
Severe LV dysfunction	0.505 (0.055)	1.657	(1.489 ; 1.845)	<0.001
LV hypertrophy	0.269 (0.082)	1.309	(1.116 ; 1.536)	<0.001
Mitral regurgitation	0.312 (0.075)	1.366	(1.179 ; 1.582)	<0.001
Pulmonary hypertension	0.165 (0.074)	1.179	(1.020 ; 1.364)	0.026
Type of treatment-				
Noninvasive		1 (ref.)		

PCI	-0.64 (0.051)	0.527	(0.477 ; 0.583)	<0.001
CABG	-1.086 (0.069)	0.338	(0.295 ; 0.387)	<0.001

SE, standard error; AdjHR, adjusted hazard ratio; CI: confidence interval; AKI, acute kidney injury; CHF, congestive heart failure; CIHD, chronic ischemic heart disease; s/p, status post; MI, myocardial infarction; PVD, peripheral vascular disease; COPD, chronic obstructive pulmonary disease; NSTEMI, non-ST-elevation myocardial infarction; STEMI, ST-elevation myocardial infarction; LV, left ventricular; PCI, percutaneous coronary intervention; CABG, coronary artery bypass graft; ref., reference group (category).
